# Concerns Oncology Clinicians Bring up During Psychodynamic Supervisions Conducted by Psycho‐Oncologists: A Qualitative Exploration

**DOI:** 10.1002/pon.70238

**Published:** 2025-08-04

**Authors:** Céline Bourquin, Amaelle Gavin, Friedrich Stiefel, Hermance Chanel, Michael Saraga, Laurent Michaud

**Affiliations:** ^1^ Psychiatric Liaison Service Lausanne University Hospital (CHUV) and University of Lausanne Lausanne Switzerland; ^2^ Service of General Psychiatry Lausanne University Hospital (CHUV) and University of Lausanne Lausanne Switzerland

**Keywords:** cancer, clinicians, content, oncology, psycho‐oncology, qualitative study, supervision, topics

## Abstract

**Objective:**

To explore the types of concern brought up by oncology clinicians to supervision sessions conducted by psycho‐oncologists.

**Methods:**

Twenty‐two audio‐recorded supervision sessions between 11 oncology supervisees, comprising nurses and medical oncologists, and 5 psycho‐oncology supervisors were selected for analysis. The method of core story creation was used to structure the supervision contents into coherent and meaningful narratives. An analysis inspired by descriptive typology was then performed on the core stories to identify patterns, group similar cases, and derive types of concern.

**Results:**

Four types of concern were identified. Type A (*I can't understand*): a sense of being lost and in need of orientation when faced with patients whose attitudes and behaviors are perceived as incomprehensible. Type B (*I understand, but can't help*): a prevailing desire for guidance in managing identified patient problems. Type C (*It's not in my hands*): feelings of powerlessness within the institutional context, with supervisees seeking to regain a sense of agency. Type D (*This moved me*): personal emotional impact of a clinical situation, prompting supervisees to reflect on their own psychological responses.

**Conclusion:**

These types of concern shed light on the psychological challenges faced by oncology clinicians and underscore the need to address them within oncology training curricula. The findings also reveal essential aspects to be integrated in psycho‐oncology training programs.

## 1 Background

Individual and group supervision conducted by psycho‐oncologists are implemented in various settings where patients with cancer are treated such as oncology and palliative care. In Switzerland, supervision is a key component of training for oncology professionals, included in both Communication Training (CT) programs [1] and advanced training in psycho‐oncology. However, access to supervision varies widely depending on multiple factors, including national healthcare systems, medical specialties and professions, clinical settings, institutional policies and work organization, the presence of liaison psychiatry, healthcare professionals' motivation, and the structure of training curricula [2, 3].

Supervision is based on a working alliance between the supervisee and the supervisor in which clinicians offer an account of their work, reflect on it, receive feedback and, where appropriate, guidance from supervisors [4]. In the oncology setting, where clinicians are faced with death and dying, supervision is viewed as an opportunity to reflect on the impact of their clinical work, their attitudes toward their own mortality, and on the emotions related to past experiences with death [5]. It is generally assumed that supervision has three main functions: formative (training), restorative (support), and normative (assessment of quality of care) [5]. Positive effects were shown on clinicians' psycho‐social competences and reflexivity, identification and understanding of interactional dynamics of the clinician‐patient encounter as well as on clinicians' emotions and unresolved issues provoked by and interfering with clinical work [6, 7, 8, 9, 10]. Moreover, supervision provides support to clinicians, and contributes to prevent stress and burnout, increases self‐confidence and work retention, and decreases feelings of powerlessness, self‐blame, and solitude [5, 11, 12, 13, 14, 15, 16].

While supervision is an essential and seemingly beneficial activity of psycho‐oncologists, empirical research on the subject is scarce [5]. For instance, little is known on the distinctive aspects of supervision in cancer care and the specific needs of clinicians [5, 17]. Studies examining the content of supervision across various settings and the topics discussed provide some insights in this direction. Outside the cancer setting, Pearce et al. [18] identified in a systematic review three recurring themes in supervision sessions with nurses and allied health professionals: reflective practice, task‐oriented content, and stress management. Lindahl et al. [19] investigated the content of group‐based supervision with nurses working in critical care and showed that participants addressed the supervision process itself, issues related to the nursing profession (degree of autonomy, organization, and relationship with patients and families), the nurse‐physician relationship (conflicts and courage to speak out), and the caring situation (delivery of care, encounters with patients or families, and feelings of unity).

Regarding themes and contents discussed in supervision with health care professionals caring for patients with cancer, only few studies exist. For example, Udo et al. [20], investigating group supervision, found that participants focused on existential dimensions of cancer and associated own emotions. Supervisees reported feelings of powerlessness, identification with patients and getting close or keeping distance. Salander and Sandström [21] identified key themes discussed in a Balint‐inspired reflective forum for oncology residents, based on the supervisor's notes. The cases presented were categorized as types of communication challenges: in the clinical relationship, in organizational matters, and with close relatives of the patient.

Based on our experience as CT trainers and individual and group supervisors of oncology and palliative care professionals, we assume that the setting of individual supervision differs from that of group supervision—which has been explored more often–, with supervisees feeling more comfortable to talk about their preoccupations without having to maintain a certain vigilance regarding what other participants might hear, think, feel or say. Against this background, our study aimed to explore the types of concern oncology clinicians address in individual supervision conducted by psycho‐oncologists. The concerns brought into supervision inform about the psychological challenges oncology clinicians encounter in their daily clinical practice.

## 2 Methods

This study used a qualitative descriptive design based on audio‐recorded individual supervision sessions conducted by psycho‐oncologists with oncology clinicians (physicians and nurses).

### 2.1 Material, Setting, and Participants

The supervision sessions were part of a corpus including, at the time of the study, 74 individual supervision sessions between 5 psycho‐oncologists and 17 medical oncologists or oncology nurses. This material was audio‐recorded in the context of two programs which include supervision as part of the training: the Swiss CT program for oncology professionals, which is mandatory for physicians [1], including 4 individual supervision sessions, and the Certificate of Advanced Studies (CAS) in psycho‐oncology for Swiss French‐speaking health care professionals caring for cancer patients, including 8 individual supervision sessions.

**FIGURE 1:**
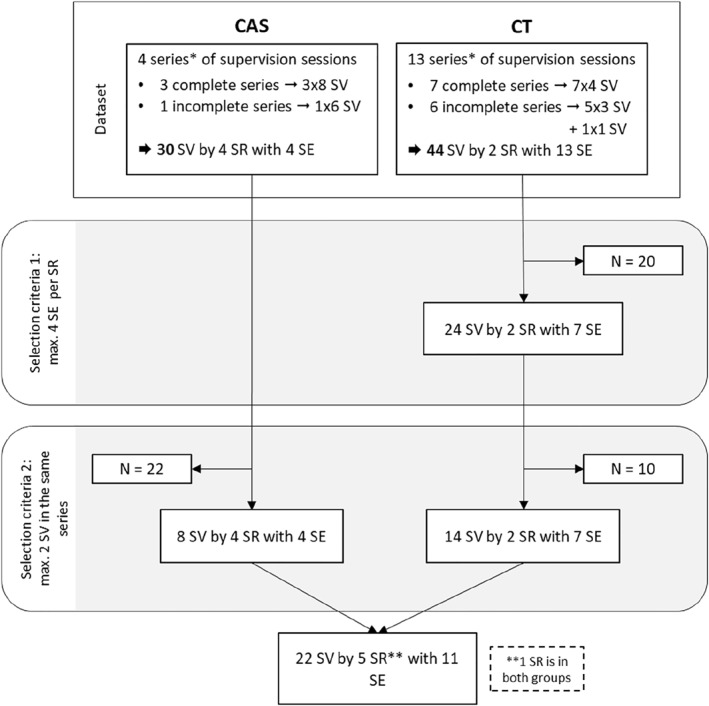
Flowchart of the selection process of the study material. *CAS participants must take part to 8 SV sessions, and CT program participants to 4. SE = supervisee; SR = supervisor; SV = supervision session.

The study material consisted of supervision sessions selected based on 2 criteria. First, to avoid including too many sessions conducted by the same supervisor, the number of supervisees per supervisor was limited to four (criterion 1). Second, to obtain a substantial sample while avoiding an overrepresentation of certain supervisees, the number of supervision sessions per supervisor‐supervisee dyad was limited to 2 (criterion 2). This process resulted in the identification of 22 supervision sessions. The second and third supervision sessions between a supervisee and a supervisor were selected whenever possible; in 4 out of 22 cases, an exception was made because the corresponding recordings were either missing or of insufficient quality to be used. This procedure ensures a certain degree of consistency, assuming that the frequency of supervisory sessions shapes the supervisor‐supervisee relationship and, consequently, the supervision process [22]. The sessions, usually planned for an hour, lasted between 28 and 67 min (median duration of 53 min) and were conducted by 3 psychiatrists and 2 psychologists with extensive experience in supervision. The supervisees were 7 oncologists and 4 oncology nurses. Oncologists, in their last year of residency, were participants of the CT program and nurses participated in the CAS in psycho‐oncology, which requires at least 5 years of practice in oncology. The flowchart in Figure 1 shows the steps for selecting the study material. Data allowing to identify individuals (e.g., patients' surname or location) appearing in the audio‐recorded supervisions were coded to protect confidentiality during the transcription process, which was carried out by an external professional transcriptionist.

Both the supervisors and the supervisees were fully informed of the aims of the study and provided written consent for the audio‐recording of the sessions and subsequent use of the data for research purposes; to preserve confidentiality, their characteristics are not presented here.

#### 2.1.1 Supervisors, Supervisees and Supervision Approach

The supervisory team consisted of three male psychiatrists and two female psychologists, four of the five supervisors had a psychodynamic orientation, while one psychologist was trained in cognitive‐behavioral therapy (CBT). All were senior staff members with over fifteen years of experience supervising somatic clinicians, and they possessed sound knowledge of both the institutional and oncological contexts. Two psychologists and one psychiatrist had worked for several years as consultants and supervisors in oncology, whereas the other two psychiatrists had worked in different medical settings.

At the time of the study, the supervisors no longer had direct clinical roles within the oncology setting and had no prior professional relationship with the supervisees. This may have helped reduce potential social desirability bias on the part of the supervisees. The supervisory approach was predominantly psychodynamic, based on basic assumptions such as transference, countertransference, intrapsychic conflict, defense mechanisms (in both patients and clinicians), the importance of early development or biography, and unconscious processes. This approach was also embraced by the CBT‐trained psychologist, who had collaborated for many years with psychodynamic supervisors as a trainer in the Swiss CT program [1]. Supervisees were invited to present a clinical situation that had raised concerns and were encouraged to describe in detail the patient's attitudes, emotions, thoughts, statements, and behaviors. Depending on the specific situation, the focus of supervision may include clinicians' personal experiences and emotional responses (including counter‐attitudes), the evolving relational dynamics between clinician and patient, the patient's psychological functioning, dyadic interaction patterns, or institutional factors such as care discontinuity or third‐party interferences (e.g., from professional hierarchies).

The nurse supervisees were experienced senior oncology professionals. Many had already participated in group supervision, and some had also received individual supervision through the Swiss CT program, which they have the possibility to join on a voluntary basis. Most physician supervisees, by contrast, were experiencing individual supervision for the first time as part of the CT program, which is mandatory for medical oncologists.

### 2.2 Data Analysis

Supervision audios and transcripts were used together throughout the analysis process, which was carried out in parallel by three of the authors (CB, AG, HC); all three authors separately examined all audio‐recordings and transcripts of the included supervision sessions and coded the material. The analysis and preliminary findings were regularly discussed within the restricted (CB, AG, HC, LM) or extended research group (all the authors). Parallel analysis of the same data by three researchers, combined with group discussion and feedback, helped to minimize bias and enhanced the credibility of the findings. The MAXQDA software (version 22.1.1) was utilized for supporting data management and analysis.

The method of core story creation [23] was used to organize supervision contents in a narrative and meaningful way. This method enables narrative reconfiguration by producing storied constructs in which key elements and events (or plots) identified in the data are thematically and temporally structured [23]. In this study, the constructs, namely, the completed core stories, were one to two pages in length, ranging from 357 to 957 words. An analysis inspired by descriptive typology [24] was then conducted on the core stories to identify patterns, group similar cases, and ultimately derive types.

Through familiarization with the material and iterative reading and listening, an initial distinction emerged between (i) the beginning of a supervision session, when supervisees presented their clinical case and related concerns without supervisors' intervention (starting content), and (ii) the subsequent elaboration and co‐construction around this material by the supervisor‐supervisee dyad (worked content). This article focuses on starting contents, the worked contents will be presented in another article together with the types of intervention used by the supervisors.

In the typological analysis, core stories were systematically compared and contrasted to identify both similarities and differences. This process facilitated the formation of groups of cases that reflected similar concerns brought up by clinicians. These groups, constituting the emergent types, were named, and comprehensive descriptions were developed, outlining their defining characteristics.

Researchers' consensus was reached for all the steps described above and for the interpretive work, which was carried out by the extended research group. This interpretive work focused on understanding the underlying meanings of the concerns expressed by oncology clinicians during supervision and the internal reactions that motivated them to bring the cases to supervision.

## 3 Results

Four different concerns of oncology clinicians emerged from the content type analysis. We named them with narrative titles, which evoke a pattern of action/reaction: *I can't understand*; *I understand, but can't help*; *it's not in my hands*; and *this moved me*. Vignettes featuring supervisee quotes are shown in Tables 1, 2, 3, 4 below to exemplify each type.

**TABLE 1:** | Vignette and quotes illustrating type A (I can't understand).

Vignette	Quotes
A male oncologist presented the case of a female patient suffering from ovarian carcinoma. After having undergone chemotherapy in Switzerland and achieving remission, the patient relocated to an eastern european country. The oncologist first met the patient after her relocation, based on her repeated requests to continue long‐distance follow‐up in Switzerland. He ultimately agreed to follow her, albeit with reservations, having expressed concerns about the potential impact on the quality of care. Consequently, the patient traveled back and forth between her country of residence and Switzerland for her care.	
During a new treatment regimen in Switzerland for a recurrence of the disease, the oncologist learned from the patient that she had also consulted a professor of gynecology of another swiss hospital who had previously been involved in her care. She had forwarded her entire medical file to him without informing the oncologist.	*During the whole treatment here, she said, ‘I already talked to the professor […], he's the gynecologist, because I had—4 years ago or maybe two—it was him who had been following me, so I sent him all the files.’ She did all that without telling me anything. I mean, I could've sent things to colleagues, of course, no problem at all, you know, if she has a preferred gynecologist, that's totally fine with me. But she did everything without saying a word to me, and so then I wrote to the professor […] I handled things with him, but yeah, in the end she's basically doing things twice.*
Upon completion of the new treatment, the patient expressed her wish to return to her country of residence and undergo specific follow‐up related to imaging there. The oncologist attempted to dissuade her, emphasizing the negative impact of variations in imaging equipment on longitudinal assessment and the risk of translation issues and interpretative discrepancies. Nevertheless, the patient maintained her position and left Switzerland.	
The oncologist reported that communication and follow‐up with this patient had proven particularly challenging. Despite his efforts to be understanding and supportive, the patient had consistently followed her own judgment rather than adhering to the recommended or advised medical guidance. She frequently questioned and renegotiated aspects of her care, including well‐established follow‐up protocols. She also duplicated efforts by involving multiple healthcare professionals and undertook diagnostic procedures on her own initiative—often one or 2 months ahead of schedule and without medical indication. She often contacted the oncologist shortly after receiving the results to inquire about them.	*She wanted to do things every month, she was really […] in constant battle each time over things that are usually pretty straightforward for follow‐up. […] She was checking the markers 2 months or a month before our appointment […] though no one had actually asked for that marker to be checked. She was the one doing it, but then she wanted to know the result.*
The oncologist had explicitly addressed these concerns with the patient. While he acknowledged the challenges she was experiencing, he still expected her to follow what he considered to be ‘the right thing to do.’ In this context, the communication was regarded as inadequate by the oncologist.	*It was a difficult communication from start to finish, because she just did whatever she wanted. Never what was actually the right thing to do […]. I can totally get that she was dealing with something hard to control, but still, I was trying to do things, take steps to help her, but they were never followed through [by the patient]. So maybe I—maybe I didn't communicate the right way, because honestly, it just wasn't good communication at all.*

**TABLE 2:** | Vignette and quotes illustrating type B (I understand, but can't help).

Vignette	Quotes
A female nurse presented a situation involving a female patient between the ages of 45 and 50 who was undergoing radiotherapy, after a complete mastectomy for breast cancer. The patient was described as eager to proceed with reconstructive surgery as soon as possible.	
The patient was reported to be in a “young” relationship, having been married for 2 years to a man she had not known for much longer before their marriage. The nurse and the husband shared a common geographical and linguistic origin, which had fostered a certain affinity between them.	
From the beginning of care, the husband had been consistently present, attending nearly every consultation. However, the nurse observed a shift in the couple's dynamic: Initially, the husband spoke more, while the patient appeared very reserved—possibly even slightly submissive, according to the nurse. Over time, the patient became more vocal, while the husband spoke less. Nonetheless, decisions were consistently made jointly by the couple, who remained actively engaged and adopted a questioning attitude throughout the care process. They refused chemotherapy and continued to question whether the mastectomy had truly been the right decision. According to the nurse, her relationship with the couple was shaped not so much by mistrust as by a fear on their part about how to position themselves in view of the treatment options.	*But it's still kind of a tense situation, even now, 5 months later, and there's a lot of, not like, mistrust, but… I feel like they're kind of scared they might've made the wrong decision [… (regarding the mastectomy)]. They refused—she refused—well, I say “they” because they always decide together. Chemotherapy was refused.*
The encounters with the couple were described as particularly unusual for the nurse, who had to make herself available and to dedicate time to the couple when one or both partners occasionally arrived without prior appointment or advanced notice, often driven by anxiety.	*These are kind of special consultations because sometimes they just show up, like that, without warning, often driven by anxiety—at least for her, and for him too, sometimes, like when he's waiting for her during radiation, he'll quickly come see me. And last week, I actually had an hour for them. I was lucky I didn't have any consultations.*
Although the nurse reported a good relationship with the couple, she still felt significantly challenged by the situation; she had the impression that the couple was negotiating with her, testing her, expecting her to prove herself and to demonstrate her competence, while also generally challenging clinical authority. The husband in particular requested access to all medical reports and frequently questioned medical opinions and decisions, despite not being a healthcare professional.	*It was really hard for me—the first consultation was really difficult because I felt like I had to sort of prove myself, like, to show that I'm competent, you know? Because he took everything, you see? He asked for all the biopsy documents,* […] *I mean, we had a good feeling right away, but still, it felt like he was really digging into things.*

**TABLE 3:** | Vignette and quotes illustrating type C (It's not in my hands).

Vignette	Quotes
A male oncologist described an interaction with a male patient in the context of a clinical trial. The patient was reported to have a highly refractory disease, with limited therapeutic options. Participation in the clinical trial required the patient to undergo a series of burdensome procedures and constraints. The oncologist reported that the patient had invested significant time and energy in the process and had placed considerable hope in the potential outcome.	*It's really heavy, all the things we ask. This patient had surgery, then a biopsy, then another biopsy. He went through all those preparatory treatments. There were other days when he traveled a long way just to do the blood filtration. It didn't work, so he came back a second time. He invested a huge amount of time with the hope this study would work out. And in the end, as part of the study process, he was hospitalized.*
The oncologist experienced considerable discomfort regarding the decision to exclude the patient from the trial after completion of chemotherapy.	*I had a… yeah, a discussion, a conversation that was really… I felt really uncomfortable in the context of this clinical trial […]*
	*And actually, in the end (laughs a little), after the chemotherapy, we decided not to go any further, to stop, and in the end, we decided not to give this treatment to the patient.*
He described the patient's reaction as one of intense anger and disappointment about how he had been managed. The oncologist considered the patient's reaction as legitimate and empathized with his profound psychological distress. The oncologist recounted that, at the time the decision was communicated, the patient almost refused to speak with him and attempted to negotiate for continuation of the trial, but without success. The patient ultimately left the hospital still very angry and with significant physical symptoms.	*He tried to negotiate. He was really angry with us. It was really complicated, and he left feeling very, very disappointed with the whole way our team had handled thing—which is… understandable, I think. But again, it was our team that made the decision, based on scientific grounds, and we just pulled the rug from under his feet at the last minute, and he's still dealing with the consequences of that.*
The oncologist reflected more broadly on the team's approach to decision‐making in the context of clinical trials. Of course, the various stages, procedures, and challenges, as well as the possibility of failure and early termination, are clearly explained to patients. These decisions are made collectively by the team based on scientific criteria, with limited consideration for the psychological impact on patients. In such circumstances, the intention to avoid harm or negative consequences can sometimes be compromised.	*It was somewhere between the… the emotions or the scientific principle that we don't want to cause harm or have negative effects on the patient—but psychologically, for this patient, it was really, really, really hard, and he was really angry. He understood that there are all these steps afterward, but once we committed to hospitalizing him and giving him the treatment now, it was kind of like—either we go all the way or we don't. […] we also have to be rigid sometimes, because we're not really taking into account the psychological impact on the patient.*
	*He was really angry that these steps hadn't been clearly defined from the start, and that it all had to be explained like that at the end. And you can't just keep changing the process along the way.*
The oncologist believed that the patient had understood the rationale and constraints of the trial. However, in his view, the fact that the patient had been hospitalized and had received chemotherapy may have given the misleading impression that the team would not reverse its decision, and that the trial agent would indeed be administered. This unexpected reversal may have contributed to the intensity of the patient's reaction.	

**TABLE 4:** | Vignette and quotes illustrating type D (This moved me).

Vignette	Quotes
A male oncology medical resident recounted a situation he wished to reflect on, involving a male patient with lung cancer and a severe health condition, who was considered a VIP. From his perspective the patient was not a “true” VIP, but rather someone with personal connections to certain professors of the hospital. The patient had requested an urgent consultation due to a skin rash following a cycle of immunotherapy. As a result, the resident saw the patient between two scheduled appointments for other patients and was working under significant time constraints.	*And I see him as an emergency between two patients, so it wasn't planned, so yeah, it's always the same thing—I mean, it's a bit like—we're kind of stressed about time. And then overall I feel like the consultation goes pretty well. A patient who's kind of—who's very anxious, who's the brother of a former top professor of the hospital, so he knows all the professors, etc. […] And so it's a patient with the VIP label, but he himself—he's not really, I mean, not really a VIP.*
According to the resident, the encounter with the patient went initially relatively well, despite the patient exhibiting marked signs of anxiety. However, later in the consultation, in the presence of the supervising oncologist, the patient suddenly began to cry, expressing his difficulties and the extent of his suffering. The resident was struck by this emotional reaction. For him, consultations generally adhered to a predictable structure: In the initial phase, residents were expected to absorb and contain patients' emotions, acting as “emotional sponges.” In the second phase, residents present the patient's case along with potential treatment options to the supervising oncologist. By that stage, it was generally assumed that the emotional reaction of patients had already passed.	*During that second part of the consultation, all of a sudden the patient, at that moment actually, he really started crying and really saying, like you know, that he was really suffering because his body was really itchy, because yeah, it was complicated. And what struck me is that I think during the whole first part of the consultation, well, I was—yeah in a way I was in a rush, I was kind of treating it like, you know, it's a skin rash, it's something expected, etc. And I was asking myself if—well I'm still kind of asking myself what I had done—I mean it's not that I would have wanted him to cry with me, but I was thinking maybe I hadn't opened that door for him.*
The oncologist therefore wondered whether he had failed to “open that door,” questioning if something in his approach might have prevented the patient from expressing his distress earlier in the consultation. He also reflected on whether he might have been too factual with the patient. Pressed for time, he approached the skin rash as a routine issue. He typically tried to maintain focus on the clinical problem, when time was limited.	
When the patient began to cry, the resident felt somewhat detached from the situation and slightly uncomfortable in the presence of the supervising oncologist. During the supervision, he sought to rationalize his reaction, attributing it partly to a mild irritation toward the VIP status of the patient. More broadly, the oncologist acknowledged feeling annoyed by VIP or “special” patients who seemed to consider themselves more important than others. Nevertheless, he emphasized that he usually made a conscious effort to put such irritation aside to remain empathetic and kind when interacting with patients.	*I realized that—even when he was crying afterward, I was kind of detached from his situation. I mean… […] maybe because he was a patient who, well, kind of annoyed me a bit, you know, because he was [VIP] and—no but yeah, it's that kind of patient who acts—who tends to—those patients who think they're more important than the others, it's always a bit of a… it's always kind of—I think that's what it was. […] I think he annoyed me a little. But usually, we try to go beyond that annoyance. Usually—I mean, sometimes the more they annoy me, the more I try to be nice to them.*

### 3.1 I Can't Understand (Type A)

Supervisees perceived patients as showing unexpected or disproportionate reactions, or as having contributed to their difficult situations. In the cases presented, patients exhibited a variety of behaviors such as for instance downplaying their condition, refusing to comply, consulting other physicians without telling them, reacting very angrily to minor modifications of treatment or procedures, or making inadequate comments. They provoked negative feelings in the supervisees, like anger, and a sense of helplessness, which were key in the supervision session. Supervisees felt also very unsettled and were confronted with their limits as they struggled to understand the situation (see Table 1).

### 3.2 I Understand, But Can't Help (Type B)

Here, the supervisees had a certain comprehension of what was going on. They felt generally empathetic toward the patients, but had mixed feelings, for instance fear of, or admiration for, the patients' behaviors. The supervisees did not know how to handle the situation and how to respond to the patients' needs, especially on a psychological level. For example, patients were identified as having a poor body image and an eating disorder, or as regressively behaving “like a big child,” which hindered their ability to adequately adapt to the disease and treatments. They distrusted the supervisees or had past traumas that seemed to be reactivated by the oncological disease (see Table 2).

### 3.3 It's Not in My Hands (Type C)

Problems identified in this type were related to the clinical practice or trials, which created a strong resonance in the supervisees without having the possibility to interfere and act upon. The cases or concerns discussed involved, for example, situations where a nurse supervisee observed a physician continuing aggressive anticancer treatment even when it no longer seemed beneficial, or where patients in clinical trials seemed not being properly cared for. The situations provoked a sense of uneasy bystanding or a feeling of loss of professional purpose among the supervisees, who were facing contextual effects beyond their influence or control (see Table 3).

### 3.4 This Moved Me (Type D)

Finally, the clinical situation or the patient stimulated supervisees to reflect on themselves, the way they relate to the world around them, and the relationship they entertain with patients. For instance, supervisees presented situations such as having to announce imminent death to a patient who was seen for the first time, caring for young dying patients, or dealing with a patient who opened up to other health care professionals but not the supervisee. The primary focus was not on the patients themselves, but on the supervisees' experiences, the challenging tasks they faced within the institution, and their psychological functioning (see Table 4).

Types D (*N* = 9) and A (*N* = 7) were more frequent in the material, compared with Type B (*N* = 4) and type C (*N* = 2).

In a nutshell, the results show 4 types of concern that oncology clinicians choose to address in supervision. In type A, concern was related to feeling lost and in need of orientation for situations considered as out‐of‐bounds; in type B, the desire for guidance on how to handle an identified problem prevailed; in type C, supervisees felt powerlessness within the institutional context and were seeking to gain some agency (capacity to act); and in type D, supervisees witnessed a situation which affected them, and prompted them to focus on themselves by taking a step back to reflect on their own psychological functioning.

## 4 Discussion

The findings highlight distinct psychological challenges faced by oncology clinicians. We will explore them drawing on our own and others' previous research, as well as our experiences in supervision and communication training.

From a general perspective, the findings show that oncology clinicians deal with concerns not only related to the clinical and scientific aspects of cancer care, but also to patients' psychosocial difficulties and suffering, institutional constraints, and their own emotional resonance and personal reflections. From a psychological standpoint, these concerns are largely linked to the experience of limits –in understanding and supporting patients, in enduring institutional constraints, and in managing one's own psychological resonances. These limits can be particularly difficult to bear in oncology, as they are also the limits inherent to the somatic perspective: the limits of cure, of treatment, and of life itself. The findings of this study may thus reflect the specificities of oncology as a “clinic of limits”. They also suggest that supervisors should have a certain familiarity with the realities of oncology care and experience in working with the notion of limits, to be valid and effective discussion partners. Importantly, supervision does not aim to overcome these limits, which is rarely possible, but rather to help clinicians learn how to engage with them without being overwhelmed by anger, guilt, or anxiety. Supervision also serves to explore what these limits provoke in each individual clinician, and to understand how and why they deal with them in the way they do. Finally, supervisors should be attentive to parallel processes, specifically the unconscious replication of the therapeutic relationship (in this case, the patient‐oncologist dynamic), within the supervisory context [25]. The way limits are handled in the patient‐oncologist relationship, for example through avoidance, may be mirrored in supervision, for example when supervisors avoid confronting supervisees about their tendency to avoid [26].

When considering the four types of concerns identified, it is important to recognize that each type encompasses heterogeneous situations, which were grouped based on the clinicians' internal reactions that motivated them to bring these cases to supervision. While the situations may differ, the clinicians' subjective experience remains the same. We chose not to explore patient difficulties in detail within each type, as the focus of this study was on the clinician's experience. Furthermore, a situation that is distressing for one clinician may not be for another.

In type A concerns, patients' reactions, behaviors, emotions, or attitudes were not understandable by oncology clinicians, who consequently experienced a range of negative feelings such as helplessness, anger, or deception. This lack of a key for understanding the psychology of patients may have various origins. First, oncology clinicians have different sensitivities, motivations, and competence to understand patients from a psychological perspective, especially when patients show psychopathology or peculiar attitudes and behaviors [27, 28]. Therefore, ‘not understanding a patient’ may depend on how psychologically minded oncology clinicians are, and/or on their motivation to take the patient's psychosocial aspects into account. Second, continuity of care [29] and knowledge of relevant biographical data [30], which could allow to understand the patient, are often lacking in the oncology setting, also due to time constraints. Third, patients may differ in their access to their inner world, as well as in their capacity‐or willingness‐to share their suffering with clinicians [31], due to psychological defenses, individual coping styles, or a lack of perceived need. They may also be reluctant to open up, either due to the oncology clinician's limited skills in fostering a therapeutic environment, or as a result of previous painful experiences with healthcare professionals, thus understandably seeking to protect themselves. This can obscure a clear understanding of how patients experience and relate to the world. In such situations, the primary psychological challenge for clinicians is to keep, gain, or regain empathy toward the patient. To do so, clinicians may rely on several elements: recognizing transference phenomena (e.g., how past attachment difficulties shape the patient's relationship with caregivers) and adopting a reflective stance toward their own reactions (e.g., countertransference shaped by their own developmental history and biography). Understanding that such situations may reflect broader dispositional patterns, on the part of either the patient or the clinician [32], can help clinicians tolerate the encounter and interpret it less personally. In cases of countertransference, it may also help them identify and work through their own difficulties that contribute to such situations. These perspectives are central to the supervision process and thus constitute key foci for supervisors.

In type B concerns, clinicians were able to recognize, at least in part, the psychic manifestations of their patients, yet their desire to help remained unfulfilled, which they experienced as both troubling and frustrating. In these situations, patients also displayed unusual reactions, emotions, behaviors, or attitudes. While clinicians were often puzzled by these expressions, they nevertheless appeared to understand their patients to some extent. They were concerned by the fact that they wished but were unable to help them. As a result, they experienced feelings of helplessness, though for different reasons than clinicians in type A concerns. In type B cases, the main psychological challenge lies in tolerating the fact that certain psychological manifestations, especially when persistent, cannot be changed through simple advice or rational discussion alone. Some patients' attitudes and behaviors may persist and resist change, as psychological traits and states frequently represent intrapsychic “solutions” that cannot simply be addressed with common sense or straightforward reasoning [33]; only a psychotherapeutic approach is likely to be effective, but it may take time. The challenge for the clinician is to address these problematic reactions, emotions, behaviors, and attitudes through a careful and comprehensive exploration. Entering the patient's inner world is not an easy endeavor, especially when they suffer a lot [34]. To do so, clinicians must overcome their own fears and hesitations in engaging with the psychological dimensions of care. Yet it is often precisely this process of exploration and shared understanding of the suffering that helps motivate patients to accept specialized psycho‐oncological support [7, 34]. With patients who resist, the challenge for clinicians is to avoid feeling guilty and to accept both the patient's and their own limits; so long as there is no immediate risk of serious self‐harm and the patient is competent to decide. This is a particularly difficult task, as medical rationality typically values problem‐solving; a rationality that do not always apply to psychiatric problems [35]. Holding back from action, and staying present with the patient, is often not perceived as being helpful, even though it is often exactly what the patient needs [35]. Early in their careers, clinicians often hear an internal voice urging, “You can't just sit here and do nothing.” With experience, however, this voice may shift to say, “Don't try to do something. Just sit here.” In this regard, the supervisor plays a key role. By modeling the capacity to tolerate uncertainty and helplessness, supervisors demonstrate that it is not only possible, but often clinically valuable to refrain from immediate intervention. Waiting, listening attentively, and offering presence can be therapeutic in themselves, as the expression and sharing of psychological pain helps patients feel less alone with their troubles. When patients resist, supervisors must help clinicians see that this resistance may arise from the patient's inner world, not from their own shortcomings. However, if supervisors identify defensive attitudes in supervisees, they should also consider helping them understand that a patient's resistance may, in some cases, reflect the clinician's own resistance to engaging with and exploring the patient's inner world.

In type C concerns, clinicians experience cognitive dissonance, often accompanied by anger, confusion, or helplessness, due to disagreement with how a patient was treated, whether in clinical practice or within research trials. In these cases, supervisees react primarily to institutional constraints and seek a sense of agency. Unlike type A and B concerns, type C concerns are rooted in a context‐specific sense of powerlessness. The medical world operates within a rigid framework of rules, hierarchies, corporatism, and conformism [36], which can give rise to moral distress. The intensity of this distress may vary according to an individual's resilience, ethical sensitivity, and the degree of perceived institutional limitations [37]. In such situations, the key psychological challenge for clinicians is to stop merely enduring and regain a sense of direction. The supervisor's main role is often to offer a third‐party perspective, to validate the supervisee's perceptions, and to provide orientation by referring to the legal, ethical, and deontological frameworks, and, when necessary, guide them to the appropriate interlocutor, such as institutional hierarchies or human resources. Based on our experience, there are additional oncology‐specific characteristics that may give rise to type C concerns. These include the often extreme physical vulnerability of patients; the use of powerful yet potentially harmful treatments; the impulse to act in the face of existential threat; prognostic uncertainty; the tension between time constraints and the desire to accompany the patient; power differentials between oncologists and patients; and the need for great prudence when delivering information. Together, these factors can contribute significantly to oncology clinicians' distress [7].

In type D concerns, clinicians resonate deeply with their patients and spontaneously speak about themselves, often questioning their own stances, and ways of being in the world. While some of these situations resemble those seen in type A or B concerns, type D is distinguished by the clinician's explicit wish to take a reflective stance. This highlights a central dimension of clinical work, particularly relevant in the oncology setting: the person of the clinician‐with their emotional history, personality, and personal values‐is directly implicated in the care process. Indeed, personal characteristics such as empathy, emotional self‐awareness, and communication style significantly influence the clinical encounter and can affect patient outcomes [38].

In these situations, the main psychological challenge for clinicians is to disentangle what belongs to the patient and what originates from themselves. At times, clinicians' resonances may reflect pure countertransference reactions, that is personal issues activated independently of the patient's actual behavior or communication. For example, a clinician may react intensively to a patient's comment, interpreting it as critical or rejecting, even when the remark was completely benign [39].

Even more challenging are transference‐countertransference interactions, in which both the clinician and the patient contribute to the emotional dynamic. Understanding these relational processes requires an awareness of one's inner world, insight into the patient's psychology, and the ability to identify the ingredients of the interactional bond. Based on such observations, we have developed a clinician‐centered supervision format for clinicians reporting type D concerns and who are motivated to work on themselves [39, 40].

## 5 Strengths and Limitations of the Study

Strengths of the study are that it goes beyond a descriptive account of themes discussed in supervision sessions. It gives voice to oncology clinicians' concerns, elicited in a context that fosters honesty and is free from common assumptions in psycho‐oncology. This approach allows to make inferences about the psychological challenges faced by oncology clinicians, and provide valuable insights into what should be taught in training of both oncology clinicians and of future supervisors working in cancer care.

There are two primary limitations to this study. First, most nurses had already participated in group supervision and, in some cases, had received individual supervision through the Swiss CT program, which they can join on a voluntary basis. In contrast, the physician supervisees were experiencing individual supervision for the first time, as part of a mandatory requirement for their specialization in medical oncology. These differences in prior supervisory experience may have influenced their respective perceptions and use of supervision and their motivation to engage in it. A second limitation relates to the fact that participants to the CAS in psycho‐oncology were certainly very sensitive to psychological aspects of cancer care and may thus not be considered as representative. However, if these clinicians have the concerns reported, there is no reason to believe that less psychologically minded oncology clinicians handle those situations better.

## 6 Clinical Implications and Future Research

The different concerns of oncology clinicians identified in this study inform about the psychological challenges they face, which should be addressed in their curriculum. Type A situations call e.g. for training in medical psychology (e.g., attachment theory, transferential reaction, and defensive styles), Type B for guidance on how to adopt a therapeutic attitude (emotional tuning, addressing interactional dynamics), Type C for raising awareness of how contextual determinants impact the experience of patients and health care professionals, and Type D for insight‐ and reflexivity‐enhancing interventions (e.g., clinician‐centered supervision [39]). These training contents and interventions for oncology clinicians have already been called for in a consensus statement on communication training [41] and the most recent ESMO Guideline on communication in cancer care [34].

## 7 Conclusion

Oncology supervisees presented four types of concerns to psycho‐oncologists, representing distinct psychological challenges for them and requiring different interventions from their supervisors. The identified types of concerns could inform the development of targeted training content within the oncology curriculum. Furthermore, both initial and ongoing education for supervisors, informed by this study findings, should contribute to a more tailored and effective supervision process. This, in turn, is expected to reinforce the positive impact of supervision sessions.

## Author Contributions

Conceptualization and funding acquisition: C.B., F.S., M.S., L.M., data curation: C.B., F.S., formal analysis: C.B., A.G., H.C., L.M., investigation: C.B., A.G., H.C., methodology: C.B., A.G., L.M., project administration, resources and supervision: C.B., L.M., validation: C.B., F.S., L.M., writing – original draft: C.B., A.G., F.S., L.M., writing – review and editing: C.B., A.G., F.S., H.C., L.M. All authors reviewed and approved the final manuscript.

## Ethics Statement

The Ethics Committee of the Canton of Vaud, Switzerland (CER‐VD) exempted the study from ethical review (Req‐2022‐00381) because it does not fall within the scope of the Human Research Act, HRA. However, participants signed an informed consent.

## Conflicts of Interest

The authors declare no conflicts of interest.
